# The dark side of immunotherapy: pancreatic cancer

**DOI:** 10.20517/cdr.2020.13

**Published:** 2020-05-11

**Authors:** Gianluca Mucciolo, Cecilia Roux, Alessandro Scagliotti, Silvia Brugiapaglia, Francesco Novelli, Paola Cappello

**Affiliations:** ^1^Center for Experimental Research and Medical Studies (CERMS), Città della Salute e della Scienza di Torino, Turin 10126, Italy.; ^2^Department of Molecular Biotechnology and Health Sciences, University of Turin, Turin 10126, Italy.; ^3^Molecular Biotechnology Center, University of Turin, Turin 10126, Italy.; ^#^The two authors contributed equally.

**Keywords:** Pancreatic cancer, immunotherapy, cancer vaccine, immune checkpoint, adoptive cell transfer

## Abstract

Since the journal *Science* deemed cancer immunotherapy as the “breakthrough of the year” in 2014, there has been an explosion of clinical trials involving immunotherapeutic approaches that, in the last decade - thanks also to the renaissance of the immunosurveillance theory (renamed the three Es theory) - have been continuously and successfully developed. In the latest update of the development of the immuno-oncology drug pipeline, published last November by Nature Review Drug Discovery, it was clearly reported that the immunoactive drugs under study almost doubled in just two years. Of the different classes of passive and active immunotherapies, “cell therapy” is the fastest growing. The aim of this review is to discuss the preclinical and clinical studies that have focused on different immuno-oncology approaches applied to pancreatic cancer, which we assign to the “dark side” of immunotherapy, in the sense that it represents one of the solid tumors showing less response to this type of therapeutic strategy.

## Introduction

Pancreatic ductal adenocarcinoma (PDAC) represents 90% of all pancreatic malignancies^[1]^, with a 5-year survival rate of 10%^[2]^. According to the American Cancer Society, the estimated number of new pancreatic cancer cases in the USA during 2020 will be 57,600 and the anticipated deaths caused by pancreatic cancer will be 47,050^[2]^. PDAC is predicted to be the second leading cause of cancer-related death by 2030^[3]^. Similar to other epithelial malignancies, a progression model from precursor lesions to PDAC has been elaborated. These lesions are unified under the collective term of pancreatic intraepithelial neoplasia (PanIN)^[4]^. Activating mutations in the KRAS proto-oncogene have an initiating function and are present in over 90% of invasive PDAC cases. Consequently, genetic and epigenetic inactivation of suppressor genes act as promoting factors: these frequently involve p16 (90%), SMAD4 (55%) and BRCA2 (10%)^[5]^. Around 50%-70% of PDAC cases carry mutations in P53, which occur at later stages of PanIN, contributing to the malignant progression of PDAC rather than its initiation^[5]^.

The median survival of PDAC patients is 4-6 months, partly because the disease usually only becomes clinically evident at a late stage, and due to its resistance to all forms of chemotherapy and radiotherapy because of its prominent desmoplastic reaction and immunosuppressive microenvironments^[6]^. Single agent gemcitabine, and chemotherapy combinations as well, such as FOLFIRINOX (irinotecan, oxaliplatin, 5-fluorouracil and leucovorin), prolong life expectancy only moderately^[7]^. Surgical resection remains the only curative option for PDAC patients but is restricted to earlier disease stages and is applicable to less than 20% of newly diagnosed patients. Considering the few benefits provided by the approved therapies and the variety of dysregulated signaling pathways in pancreatic cancer, targeted therapies have emerged as a potential way to strategically direct treatment to tumor cells and increase the patient’s survival. Immunotherapy is another important non-chemotherapeutic strategy, and it is a treatment designed to induce or enhance the immune system response against cancer. Accordingly, the goal of immunotherapy is to stop or slow down cancer cell growth and metastatic events through the enhanced recognition of cancer cells by the host immune system^[7]^.

**Figure 1:**
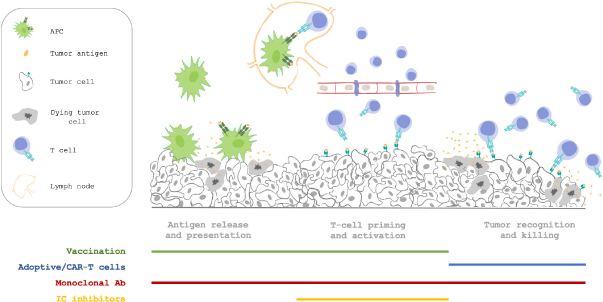
Immunotherapy intervention in the different steps of antitumor response. In this review, we will describe the current landscape of preclinical and clinical advances made in immunotherapy applied to pancreatic cancer. APC: antigen-presenting cell; CAR: chimeric antigen receptor

In agreement with the concept of the “cancer immunity cycle”^[8]^, the wide spectrum of cancer immunotherapies can be associated with each different step. Accordingly, dendritic cells (DCs) must capture neoantigens released by necrotic and apoptotic cancer cells (step 1) and present them to T cells (step 2) to induce effector T cell priming and activation against cancer-specific antigens (step 3). Activated T cells then travel through blood vessels (step 4) to infiltrate the tumo area (step 5). Here, T cells recognize specific antigens presented in association with major histocompatibility complex (MHC) class I molecules on the cancer cell surface (step 6) and kill their targets (step 7)^[8]^. The first steps inducing priming, clonal expansion and activation of immune cells can be facilitated by cancer vaccines, which play an active role in directing the patient’s own immune system towards tumor-specific antigens. In addition, adoptive immunotherapy acts in a passive way, delivering ready-to-use lymphocytes able to infiltrate tumor sites, recognize cancer cells and destroy them. Finally, monoclonal antibodies (mAbs) and immune checkpoint inhibitors (ICIs) can be designed to target different steps throughout the whole cycle, acting as potent adjuvant agents^[8]^ [Figure 1].

## Cancer vaccines

Cancer vaccines have emerged to be very promising for treating different cancer types such as melanoma and lung, breast and renal cell carcinoma^[9]^. However, vaccine-based immunotherapy in PDAC has shown controversial results, thus requiring further studies^[9]^. Vaccines are designed to prime the patient’s own T cells against cancer-specific antigens by promoting effective anticancer immunity [Figure 1]. The most effective vaccines include different combinations to overcome the immunosuppressive microenvironment promoted by the tumor itself.

Vaccines deliver antigens either in the form of DNA, peptides or tumor cells that are captured by antigen-presenting cells (APCs). Once activated, loaded DCs migrate to the nearest draining lymph nodes and present antigens on MHC molecules that are recognized by T cells through their specific T cell receptors (TCRs). These crucial steps lead to the priming and activation of T cells. One specific form of vaccine is represented by antigen-pulsed DCs that directly migrate to the lymph nodes, thus avoiding the first steps of antigen expression and capture. Hence, activated antigen-specific T cells can recirculate and migrate into the tumor microenvironment (TME), where they can again recognize specific antigens expressed by tumor cells and thus destroy them. Consequently, dying cancer cells release new antigens that trigger adaptive immune responses and allow the expansion of other cancer-specific T cells. To be effective, it is crucial that the choice of antigen be restricted to the tumor or only minimally expressed by other tissues. Indeed, genes coding for tumor-associated antigens (TAAs) can also be found in normal cells but are aberrantly expressed in cancer cells or the encoded proteins have undergone abnormal posttranslational modifications during carcinogenesis.

Significant efforts have been made to identify new pancreatic cancer-associated antigens, preferably shared by the majority of patients, such as carcinoembryonic antigen (CEA)^[10]^, mucin-1 (MUC-1)^[10]^ and the product of mutated KRAS^[10]^, to develop targeted vaccination strategies. More recently, novel TAAs have been associated with PDAC, including alpha-enolase (ENO1)^[11-14]^, carcinoembryo antigen-related cell adhesion molecule 6 (CEACAM6)^[15]^ and mucin-4 (MUC-4)^[16]^, although they are not yet in clinical use.

## GVAX (granulocyte-macrophage colony stimulating factor expressing vaccine)

One of the most promising vaccines in PDAC treatment is GVAX, a whole-cell vaccine consisting of allogeneic human pancreatic cancer cells engineered to express granulocyte-macrophage colony stimulating factor (GM-CSF)^[17]^. Several studies have highlighted that patients with advanced cancer have limited macrophage function, which could be restored by GM-CSF. Indeed, GM-CSF enhances DC activity and promotes antigen presentation to T cells^[17]^. GVAX was designed on the basis of preclinical data demonstrating that irradiated B16 melanoma cells expressing GM-CSF promoted the establishment of a long-lasting and specific antitumor response^[17]^. A phase I trial was conducted in surgically resected PDAC patients to assess its safety and effectiveness in inducing an antitumor immune response^[18]^ [Table 1]. At 8 weeks after pancreaticoduodenectomy, 14 patients received different doses of GVAX and a 6-month course of adjuvant radio- and chemotherapy. Afterwards, 6 out of 14 patients received additional vaccination. Since no dose-limiting toxicities were reported and the antitumor immunity increased in a dose-dependent manner, a follow-up phase II adjuvant study (NCT00084383) was conducted in 60 patients with resectable PDAC [Table 1]. GVAX was administered five times before 5-FU-based chemoradiotherapy and the combined treatment was administered monthly in disease-free patients for a total of four rounds^[19]^. Finally, a 5th and final dose was administered after 6 months. Data from this study showed that GVAX elicited the activation and expansion of tumor-specific CD8^+^ T cells, thus improving the patients’ OS. Interestingly, disease-free patients after the final GVAX treatment could elicit antigen spreading against a wide range of TAAs, suggesting that GVAX immunotherapy can be used both alone as an adjuvant therapy and/or in combination with conventional treatment^[19]^.

**Table 1:** Summary of clinical trials for the indicated immunotherapy approaches

Immunotherapeutic treatment	ID number	Phase	No. of patients	Cohort composition	Strategy	Results
Whole cell-based vaccination	[18]	I	14	Surgically resected	GVAX + chemoradiotherapy	No toxicities; dose-dependent antitumor immunity increased
	NCT00084383^[19]^	II	60	Surgically resected	GVAX + chemoradiotherapy	Expansion of CD8^+^ T cells; increased OS
	[20]	Pilot study	50	ADV	GVAX + cyclophosphamide	Minimal toxicities; increased T cell response; improved OS
	NCT00727441^[21]^	II	87	Surgically resectable	GVAX ± cyclophosphamide	TLS formation; increased T cell response; increased OS
	NCT00585845^[23]^	I	17	Treatment-refractory	CRS-207	Dose-dependent antitumor immunity increased
	NCT01417000^[24]^	II	93	Pretreated metastatic	GVAX + cyclophosphamide ± CRS-207	Minimal toxicities; increased T cell response; increased OS
	NCT02004262^[25]^	IIb	213	Pretreated metastatic	GVAX + cyclophosphamide + CRS-207; CRS-207 alone; CT	GVAX + CT + CRS-207 no improved survival over CT
Peptide-based vaccination	[27]	I/II	5	ADV	Synthetic RAS-loaded APCs	2/5 pts showed increased immune response and OS
	CTN-95002/97004^[28]^	I/II	48	10 resected/38 ADV	Synthetic RAS-loaded APCs + GM-CSF	Increased OS and immune response in 58% of pts
	CTN-95002/98010^[29]^	10 yrs follow-up	23	Surgically resected	Synthetic RAS-loaded APCs + GM-CSF	Long-lasting vaccine-induced immune response in 85% of pts
	[31]	I/II	48	Unresectable	GV1001 + GM-CSF	Vaccination well tolerated; immune response in 75% of pts
	ISCRCTN-4382138^[32]^	III	1062	ADV/Metastatic	CT; GV1001 after or with CT	No differences between CT and combined treatment
	UMIN000000905^[35]^	I	6	Unresectable ADV	Survivin-2B80-88 + IFN-α	> 50% of pts with positive clinical and immunological responses
	UMIN000012146^[36]^	II	83	HLA-A24^+^	Survivin-2B80-88 ± IFN-β	Combined treatment increased pts survival w/o toxicity
Dendritic cell-based vaccination	[41]	I/II	10	Surgically resected	MUC1 peptide-loaded DCs	No toxicity; 3/10 pts alive after 4 yrs
	06DZ19009^[42]^	I	7	Metastatic	MUC1 peptide-loaded DCs	No toxicity; no clinical benefits
	UMIN000004855^[44]^	I	10	HLA-A2*2402	WT1 peptide-loaded DCs	No toxicity; increased survival only in pts w/o liver metastasis
	UMIN000004063^[45]^	I	10	ADV	WT1 peptide-loaded DCs ± GEM	No toxicity; increased OS and PFS in 50% of pts
	NCT01410968^[47]^	I	12	Metastatic	hTERT/CEA/Survivin peptide loaded DCs + poly (IC:LC)	Fatigue and/or flu-like symptoms; 4/12 pts SD and 4/12 pts PD
Adoptive cell transfer	NCT00965718^[75]^	II	43	Surgically resected	MUC1-CTLs + GEM	Reduction of liver and local recurrences
	[77]		20	Treatment-refractory	CIK cells ± GEM	No differences observed between CT and combined therapy
	[78]		58	Pretreated ADV	CIK cells ± S-1	No differences observed between CT and combined therapy
CAR-T	NCT01897415^[109]^	I	6	Treatment-refractory	Anti-MSLN CAR-T	2 pts with SD; MAV tumor lesions stable in 3 pts and decreased in 1 pt by 69.2%
Monoclonal antibody	NCT00711191^[154]^	I	22	CT-naïve ADV	CP-870,893 + GEM	4 pts PR; 11 pts SD
Immune checkpoint inhibitors	NCT00112580^[177]^	II	27	ADV/metastatic	Ipilimumab (anti-CTLA-4)	1/27 subject showed a significant delayed response
	NCT00729664^[180]^	I	14	ADV	BMS-936559 (anti-PD-L1)	No response observed
	NCT01876511^[181]^	II	8	MMR-deficient	Pembrolizumab (anti PD-1)	2 CR; 3 PR; 1 SD
	NCT00556023^[187]^	Ib	34	ADV	GEM ± Tremelimumab (anti-CTLA-4)	6% PR; 21% SD; OS 7.4 months
	NCT01473940^[188]^	Ib	21	Unresectable ADV	GEM ± Ipilimumab (anti-CTLA-4)	Safe and tolerable regimen; 14% ORR; 33% SD; OS 6.9 months
	NCT023311251^[189]^	Ib/II	17	Pretreated/CT naive	Pembrolizumab (anti-PD-1) + GEM/nab-P	25% PR; 67% SD; OS 15 months (CT naïve pts); no PD pts
	NCT02309177^[190]^	I	42	CT-naïve ADV	Nivolumab (anti-PD-1)/nab-P ± GEM	2% CR; 16% PR; 46% SD; OS 9.9 months
	[197]	I	30	Pretreated ADV	Ipilimumab (anti-CTLA-4) ± GVAX	OS 5.7 months in pts with combined therapy, compared to 3.6 months in pts treated with ipilimumab alone

Pts: patients; PD: progressive disease; TLS: tertiary lymphoid structures; Yrs: years; PR: partial response; MAV: metabolically active volume; OS: overall survival; CR: complete response; CT: chemotherapy; PFS: progression-free survival; ORR: objective response rate; GEM: gemcitabine; SD: stable disease; ADV: advanced; Nab-P: nab-paclitaxel; MMR: mismatch repair; CTLA-4: cytotoxic T-lymphocyte ntigen 4; MSLN: mesothelin; CIK: cytokine-induced killer; CEA: carcinoembryonic antigen; DCs: dendritic cells; APCs: antigen-presenting cells; GM-CSF: granulocyte-macrophage colony stimulating factor; CAR: chimeric antigen receptor

To further assess the efficacy of the combination of another chemotherapeutic drug and GVAX, a study was conducted administering two allogeneic GM-CSF-secreting pancreatic cell lines (CG8020/CG2505), alone or with cyclophosphamide, in 50 patients with advanced PDAC^[20]^ [Table 1]. Again, data showed minimal treatment-related toxicity and higher specific T cell responses in patients who received the combined treatment compared to those who received vaccine or cyclophosphamide as monotherapy, resulting in improved overall survival (OS)^[20]^.

A subsequent neoadjuvant and adjuvant clinical trial (NCT00727441) was conducted in 87 resectable PDAC patients to compare the efficacy of GVAX alone or in combination with low-dose cyclophosphamide to study how the TME is altered by immunotherapy^[21]^ [Table 1]. In most patients, combined treatment caused the formation of intratumoral tertiary lymphoid aggregates, as a regulatory structure of adaptive immunity. Moreover, combined treatment showed improved survival and an increased specific T cell response^[21]^. Regarding combined GVAX and cyclophosphamide treatment, an ongoing phase II clinical trial (NCT01088789) is now in the recruiting step and will be completed by 2023. The aim of this study is to assess the safety and viability of a long-term GVAX vaccination alone or in combination with cyclophosphamide in surgically resected pancreatic adenocarcinoma. This trial will evaluate the effect of GVAX vaccination administered every 6 months for a period of 10 years.

In a different approach, the bacterium *Listeria monocytogenes*, due to its ability to induce potent innate and adaptive immunity, was engineered to express the human mesothelin TAA (CRS-207) and used as a cancer vaccine^[22]^. A phase I study (NCT00585845) was conducted in 17 treatment-refractory PDAC patients, in which safety, toxicity and clinical activity were evaluated [Table 1]. Results showed that CRS-207 was safe and tolerable, and that the immune response increased in a dose-dependent manner^[23]^. These promising results prompted investigators to combine the CRS-207 vaccine with GVAX. A phase II clinical trial (NCT01417000) was conducted in 93 previously treated patients with metastatic PDAC^[24]^ [Table 1]. Half of patients received two doses of GVAX with low-dose cyclophosphamide (Cy/GVAX) followed by four doses of CRS-207 every 3 weeks, while the second half of patients received six doses of Cy/GVAX every 3 weeks. The results demonstrated that combined treatment significantly enhanced OS, inducing a mesothelin-specific T cell response, with minimal toxicity^[24]^. However, the ECLIPSE Study (NCT02004262), a phase IIB clinical trial conducted in 303 patients, in which Cy/GVAX plus CRS-207, CRS-207 alone, and single-agent chemotherapy were compared in previously treated patients with metastatic pancreatic adenocarcinoma, suggested that the combined treatment (even if well-tolerated) did not ameliorate survival compared to chemotherapy alone^[25]^ [Table 1].

### Peptide-based cancer vaccines

In addition to cell lines, mutated peptides have also been used to immunize pancreatic cancer patients to enhance tumor-specific T cell responses. Peptide-based vaccines represent a promising tool for immunotherapy. Peptides are easily synthesized on the basis of the most immunogenic portions of TAAs or specifically identified mutations that are associated with each patient (personalized vaccine). The advantage of a peptide-based vaccine compared to the whole protein is to increase the number of peptide-MHC complexes available for T cell recognition and improve the immune response.

The most well-known driving mutation is on codon 12 of the *KRAS* gene, which has therefore been employed in a peptide-based cancer vaccine. Indeed, pioneer clinical studies assessed the safety and feasibility of synthetic RAS peptides loaded *ex vivo* on autologous antigen-presenting cells in five PDAC patients^[26,27]^ [Table 1]. Two clinical trials (CTN-95002 and CTN-97004) exclusively based on peptides, were conducted in 48 PDAC patients by combining synthetic mutant RAS peptide vaccination with GM-CSF^[28]^ [Table 1]. The results showed that specific T cell responses and increased survival were induced in almost 58% of evaluable patients. Furthermore, to evaluate potential long-term response, a follow-up study was conducted (CTN-95002 and CTN-98010)^[29]^ [Table 1]. A total of 23 patients were followed for more than 10 years and 85% of them showed a long-lasting vaccine-induced immune response, underlining the importance of combined treatments in patients carrying RAS mutations^[29]^.

Regarding tumor-specific antigens, the telomerase reverse transcriptase (TERT) protein was shown to be overexpressed by the majority of pancreatic cancers^[30]^. Based on this TAA-like feature, the GV1001 vaccine, consisting of 16 amino acids derived from human TERT, was developed^[31]^. To assess vaccine safety, stability and toxicity, a phase I/II study was conducted in 48 unresectable PDAC patients receiving scalar doses of the vaccine in combination with GM-CSF. As well as being well-tolerated, the vaccine induced a specific immune response in 75% of patients^[30]^ [Table 1]. These promising results paved the way to design the first phase III clinical trial based on GV1001 administration, called TeloVac (ISRCTN-4382138)^[32]^ [Table 1]. A total of 1062 patients with locally advanced or metastatic PDAC were subdivided into three groups, namely patients receiving gemcitabine and capecitabine, those receiving gemcitabine + capecitabine followed by GV1001 vaccination (sequentially), and those receiving gemcitabine + capecitabine and GV1001 vaccination (concurrently). Unfortunately, OS was not significantly different between chemotherapy alone and the combined treatments. In addition, it was shown that gemcitabine and capecitabine combined with GV1001 vaccination did not reduce C-reactive protein, IL6 or GM-CSF levels^[33]^. Despite the discouraging results, the study of a large number of patients enrolled in the study resulted in the identification of 19 cytokines that were significantly reduced in patients receiving sequential chemo-immunotherapy but not in the group treated concurrently with GV1001 and chemotherapy. These cytokines could potentially be used as biomarkers in clinical follow-up^[34]^.

Several studies have also highlighted survivin as one of the most promising candidates for tumor-specific immunotherapy in pancreatic cancer. Survivin is a member of the inhibitor of apoptosis protein family that prevents apoptotic cell death and its expression is high in numerous tumors, including PDAC. In particular, an HLA-A24-restricted antigenic peptide, survivin-2B80-88 (AYACNTSTL), was identified to develop a peptide-based vaccine. A phase I clinical trial (UMIN000000905) was conducted in unresectable advanced HLA-A24-positive PDAC patients, who received survivin-2B80-88 peptide in combination with interferon alpha (IFN-α)^[35]^ [Table 1]. Encouraging results emerged from the study, as more than 50% of patients had positive clinical and immune responses with stabilization of the disease. Despite the clinical results, some IFN-α-related side effects were detectable. Therefore, a phase II clinical trial (UMIN000012146) in which 83 HLA-A24-positive PDAC patients were vaccinated with survivin-2B peptide alone or in combination with interferon beta (IFN-β) was performed^[36]^ [Table 1]. Patients receiving the combined treatment had longer survival with no notable adverse effects, highlighting the reduced toxicity of IFN-β. Furthermore, immunohistochemical analyses revealed a strong infiltration of CD8^+^ T cells in lesions of vaccinated patients and a high rate of programmed cell death protein (PD-1), revealing the potential for a new combination of survivin-2B peptide-vaccine with immune checkpoint inhibitors^[37]^.

Another emerging target for immunotherapy is gastrin, a gastric hormone able to bind to the CCK-B receptor, which is overexpressed in numerous types of gastrointestinal cancers, including pancreatic cancer. Recently, a gastrin-specific peptide-based vaccine, called polyclonal antibody stimulator, was assessed for its therapeutic potential in a transplantable PDAC mouse model. It was demonstrated to elicit both a humoral and cellular immune response, with the generation of specific target antibodies and T cell activation, respectively. Furthermore, the vaccine was effective alone, and also in synergy with a PD-1 antibody. In particular, combined therapy decreased tumor burden, fibrosis and immunosuppressive elements of the TME such as regulatory T lymphocytes (Tregs) and macrophages^[38]^.

A more tailored approach based on personalized peptide vaccination strategies has also been developed for PDAC patients at the M.D. Anderson Cancer Center in collaboration with the National Cancer Institute and applied in a phase I clinical trial (NCT02600949). This approach relies on the availability of tumor samples and dedicated on-site technology. To date, however, there are no posted results that confirm that this this strategy is truly effective.

### Dendritic cell-based vaccines

Pancreatic cancer is characterized by a limited number of functional DCs, which impairs the mounting of an effective antitumor response. DCs are professional antigen-presenting cells with a pivotal role in priming T cells. Thus, *in vitro* generated mature DCs can be used to develop a cancer vaccine, aimed at increasing antigen presentation, enhancing T cell priming and expanding antigen-specific T cells. DC loading can be achieved with different sources of tumor antigens such as tumor lysates, whole tumor cells, synthetic peptides and purified proteins or transfection with mRNA or cDNA^[39]^.

One investigated target has been MUC-1, a PDAC-associated antigen that correlates with poor prognosis, metastasis and chemoresistance^[10]^. Since the MUC-1-targeted vaccine induced antitumor immunity in murine pancreatic cancer models^[40]^, its potential in inducing specific antitumor immune responses was assessed in different clinical trials. A phase I/II clinical trial evaluated the therapeutic potential of DCs loaded with MUC-1 peptide in ten surgically resected PDAC patients. No toxicity was observed, and patients were followed for more than 4 years, after which three patients were still alive without recurrences^[41]^ [Table 1]. The same approach was used in another phase I clinical trial (06DZ19009), in which seven patients with metastatic disease received the MUC-1-targeted vaccine^[42]^ [Table 1]. A specific immune response was reported, and there was no toxicity, but there were unfortunately no clinical benefits. These studies indicate MUC-1 as a promising tumor antigen, which requires further investigation^[42]^.

Another PDAC-associated antigen is the Wilms tumor gene (*WT1*), which is highly expressed in more than 75% of PDAC cases and has been reported to be highly immunogenic. For investigating the therapeutic potential of WT1, DCs were loaded with MHC class I- and/or II-restricted peptides. A retrospective analysis was conducted in 225 Japanese patients with pancreatic cancer refractory to standard treatment who received chemotherapy combined with MHC class I-restricted WT1 peptides. Interestingly, the median survival time was prolonged to 16.5 months, and WT1-targeted vaccination combined with standard chemotherapy was shown to be feasible and safe^[43]^. Another phase I pilot study (UMIN000004855) was conducted in ten PDAC patients who were treated with DCs loaded with MHC-I-restricted WT1 peptides combined with gemcitabine [Table 1]. The therapy was feasible, safe and well-tolerated; however, only patients without liver metastasis and high levels of inflammatory markers benefited from the treatment^[44]^. The phase I trial (UMIN000004063), instead used DCs loaded with both MHC class I- and MHC class II-restricted WT1 peptides combined with gemcitabine in advanced PDAC patients [Table 1]. The combined therapy was well tolerated. Half of the patients showed specific T cell responses, increased OS and progression-free survival. Interestingly, among the non-responsive patients with the combined treatment, increased levels of interleukin (IL)-6 correlated with poor prognosis^[45,46]^.

In a pilot study (NCT01410968), Mehrotra *et al.*^[47]^ evaluated the feasibility and safety of a DC-based triple vaccine: autologous HLA-A2-restricted DCs of 12 HLA-A2+ patients with unresectable or metastatic PDAC were pulsed for an hour with hTERT, CEA or survivin peptides [Table 1]. DCs were administered intradermally three times, followed immediately by intramuscular administration of a TLR3 agonist (polyinosinic:polycytolitic acid stabilized with polylysine and carboxymethylcellulose; poly IC:LC), as an adjuvant to boost T cell antitumor activity. Treatment was well tolerated, with some cases of fatigue and flu-like symptoms such as fever, night sweats and myalgia. A total of eight patients completed the study, half of them with stable disease and the other half with progressive disease. Compared to the control cohort treated with a second-line chemotherapy, which had a median survival of 4.9 months, the eight patients undergoing vaccination showed an increase in median survival by 2.8 months^[47]^.

### DNA vaccines

In the broad cancer vaccine landscape, DNA vaccination has emerged as a promising immunotherapeutic approach due to its stability and safety^[48]^. Two main findings have triggered the development of antitumor DNA vaccines, namely the ability of mammalian cells to express genes encoded by DNA plasmids and the *in vivo* humoral and cellular immune response obtained against the plasmid products^[49,50]^. An important benefit of DNA vaccines is that the antigen can be presented by both MHC class I and MHC class II, which can activate CD4^+^ and CD8^+^ T cells, and also possibly deliver several antigen genes in the same construct to obtain a wider specific immune response^[51]^.

Our laboratory is focused on identifying new antigens to employ in DNA vaccination strategies for PDAC treatment^[52]^. In particular, we have identified the glycolytic enzyme ENO1 as a promising PDAC TAA by showing that antibodies against ENO1 are detected in more than 60% of PDAC patients^[53]^. Notably, this antibody response is absent or low in healthy donors and also in patients with non-pancreatic cancer and chronic pancreatitis^[14]^. ENO1 plays a critical role in cancer proliferation, metastasis and spreading, thus therapies targeting ENO1 may be effective in hindering tumor progression^[54]^. A preclinical study demonstrated that the ENO1 DNA vaccine elicited an integrated antitumor immune response in PDAC^[55]^. In particular, the ENO1-vaccinated PDAC mouse model showed a specific cellular response that significantly prolonged survival, as well as a higher amount of anti-ENO1 immunoglobulin (IgG) and a reduction of myeloid-derived suppressor cells (MDSCs) and Tregs. This correlated with a reduced amount of PDAC lesions in ENO1-vaccinated 8-month-old tumor-bearing mice^[55]^.

An alternative DNA-vaccination method is being used in a phase I study to evaluate the safety and immunogenicity of an integrated vaccine comprising neoantigen plus mesothelin (MSLN) epitopes in PDAC patients following surgical resection and adjuvant chemotherapy (NCT03122106). The DNA vaccine combines prioritized neoantigens plus MSLN epitopes in a pING plasmid, which is administered with an electroporation device. Hopefully, neoantigen DNA vaccines will be safe and capable of generating neoantigen-specific CD4^+^ and CD8^+^ T cell responses, which will be evaluated by ELISPOT and multiparametric flow cytometry.

DNA-based approaches also include all those involving transfection of tumor cells or dendritic cells with plasmids encoding TAAs, cytokines, co-stimulatory molecules and neoantigens, which are discussed below.

### Other alternative approaches

Neoantigens are tumor-specific antigens that are not expressed by normal cells, which result from *de novo* gene alterations that lead to potentially immunogenic peptides showing MHC molecules^[56]^. Neoantigen-specific immune responses do not follow central and peripheral tolerance laws and do not affect healthy tissues. Neoantigen-based immunotherapies are patient-related and, therefore, defined as tailored-made therapies. Mutated peptides are identified by whole exome sequencing and the most immunogenic epitopes matching MHC with peptide binding prediction algorithms, i.e., NetMHC^[57]^. The whole process relies on the availability of tumor samples, and therefore, this type of approach can be applied only to surgically resected patients. Nowadays, personalized neoantigen-based vaccines are being tested in phase I clinical trials with the purpose of assessing their safety and effectiveness in pancreatic cancer patients following surgical resection and adjuvant chemotherapy (NCT03122106; NCT03558945; NCT03956056), alone or combined with programmed cell death protein 1 ligand (PD-L1) inhibitors (NCT04161755). These studies are still in the recruiting phase.

The exosome-based vaccine is another innovative immunotherapy strategy. Exosomes are small, non-toxic, and non-immunogenic endosomal-derived vesicles secreted in the extracellular space that can carry lipids, proteins, RNA or DNA. Due to their phospholipid membrane and small size, their content is protected from degradation and able to penetrate natural barriers^[58]^. Exosomes can be obtained from the patient’s own cells, thus avoiding allograft reaction and can be engineered to carry specific tumor antigens to directly target cancer cells only. Taking these advantages into account, Xiao *et al.*^[59]^ assessed the effectiveness of vaccination with tumor exosome-loaded dendritic cells in PDAC-bearing mice. Specifically, DCs loaded with PDAC-derived exosomes were administered intravenously in mice, either alone or in combination with gemcitabine, all-trans retinoic acid and sunitinib (tyrosine kinase inhibitor). Data showed that the vaccine effectively prolonged survival by inhibiting metastases and reducing the presence of MDCSs in the tumor infiltrate.

Another approach relies on virally-infected cell vaccines, replicating oncolytic viruses that are natural or genetically-modified viruses with a specific tropism for cancer cells, which generate a specific T cell activation. In this type of vaccination, cancer cells are pre-infected with tropic viruses and infused. In particular, Lu *et al.*^[60]^ generated a virus-infected reprogrammed somatic cell-derived tumor cell vaccination (VIReST), consisting of murine fibroblasts transduced with lentiviruses to obtain induced pluripotent stem cells (iPSCs). These cells were then transduced to express mutated KRAS-G12D and p53-R172H, and then differentiated into pancreatic tumor cells. Cells obtained in this way were then infected with either oncolytic adenovirus (AdV) or vaccinia virus (VV). The authors showed how the order of viruses used to infect cells was fundamental for obtaining correct antitumor activity; mice spontaneously developing PDAC were first immunized with AdV-infected mitomycin C-treated cancer cells, followed by VV-infected cancer cells after 4 weeks in a prime-boost regimen. The importance of the order is due to the characteristics of the viruses; AdV is an extremely effective Toll-like receptor (TLR) activator, while the wide range of immunomodulatory proteins expressed by VV can boost T cell responses. VIReST was able to extend the lifespan of mice by 51%, halving the percentage of mice with detectable tumors at 6 weeks. Moreover, the authors detected an increase in CD8^+^ and CD4^+^ inside tumors, together with central memory populations. Importantly, no signs of autoimmunity were detected (colitis, ileitis and antinuclear antibodies). However, the protocol was not able to completely prevent tumor progression, indicating the need to combine this approach with other strategies^[60]^.

## Adoptive cell transfer

Another important trend in cancer immunotherapy focuses on T cell-mediated activity, namely adoptive autologous T cell transfer (ACT) and chimeric antigen receptor (CAR) cells. The fundamental purpose of autologous ACT is to generate a vigorous immune-mediated antitumor response via the infusion of *ex vivo* expanded or engineered leukocytes^[61]^ [Figure 1]. Tumor infiltrating lymphocytes (TILs) isolated from freshly resected tumors were the first source of T cells for ACT. However, more recently, peripheral blood mononuclear cells (PBMCs), tumor-primed draining lymph nodes, and iPSCs have also been used^[62]^. TIL generation techniques vary slightly in each institution, but small fragments are generally kept in culture medium supplemented with IL-2 and other cytokines and cells are harvested from supernatants over 2 to 3 weeks^[63,64]^. Generally, ACT is carried out with a mix of CD8^+^ and CD4^+^ T cells, depending on the patient’s T cell ratio and their expansion *ex vivo*, but certain protocols involve defined ratios, based on indications that this may lead to more favorable outcomes^[65]^. Even if CD8^+^ T cells are more powerful in exerting their antitumor effect through secreted cytokines and direct tumor cell killing, studies have demonstrated that CD4^+^ T cells play a pivotal role in these phenomena^[66-68]^. TILs have to be extensively cultured *ex vivo* and this can result in terminally differentiated cells that no longer exert their effect when re-infused^[69]^. The best responses to ACT therapy were in metastatic melanoma patients, in which substantial results were long-lived, and the majority of patients achieved a disease-free state for many years after treatment^[61]^. In other tumor types, even if promising preliminary results had been reported, the majority of clinical responses were transitory, ultimately leading to tumor relapse. This is probably due to the limited survival of the reinfused cells and their exhaustion^[70]^. To overcome these difficulties, immune checkpoint-blocking antibodies seem to enhance ACT activity, as reported below^[71]^.

Concerning PDAC, one of the first targeted strategies applied to ACT exploited TP53 mutations present in cancer cells. This preclinical study proved that p53 peptide-specific cytotoxic T lymphocytes adoptively transferred in immunocompromised mice, were effective in suppressing PDAC growth *in vivo* and prolonged survival^[72]^. Other strategies focus on telomerase, a reverse transcriptase used to prevent senescence and maintain chromosomal stability, reported to be overexpressed in a significant percentage of PDAC (85%-90%) and considered a marker of poor prognosis^[73]^. For instance, adoptive telomerase-specific T cells obtained from peptide-immunized donor mice and stimulated with either IL-2, IL-15, APCs or allogeneic DCs, significantly slowed tumor growth and metastasis^[74]^.

One of the first PDAC-associated targets investigated in clinical trials was MUC-1. Autologous PBMCs from resectable pancreatic cancer patients obtained either before surgery or during gemcitabine treatment, were cultured with MUC-1-expressing pancreatic cancer YPK-1 cells for 10 days and re-infused in the patients for a total of three rounds^[75,76]^ [Table 1]. It was shown that post-surgery gemcitabine-based regimens combined with adoptive transfer of MUC-1-specific cytotoxic T cells reduced the incidence of local recurrence and liver recurrence as well (33% compared to 60%)^[75]^. Overall, this type of therapy had no adverse events attributable to immunotherapy and could thus be a potential therapeutic strategy for pancreatic cancer.

Cytokine-induced killer (CIK) cells are another source of cells for ACT. CIK cells are generated from peripheral blood lymphocytes and exert an HLA-unrestricted antitumor activity. In a phase II trial (NCT00965718), advanced PDAC patients under gemcitabine regimens received adoptive transfer of CIK cells as a second-line therapy^[77]^ [Table 1]. Another study tried to combine S-1 (an oral fluorouracil anticancer drug comprising tegafur, gimeracil, and oteracil potassium) with or without CIK in advanced PDAC patients who previously received gemcitabine as a first-line therapy^[78]^ [Table 1]. Unfortunately, both studies showed a limited effect of CIK-based therapy in improving patient survival and disease control rate.

As ACT is more widely used, it is crucial that all centers be aware and become familiar with managing adverse events such as cytokine release syndrome, which may be primarily related to the preconditioning regimen, particularly to the administration of high doses of IL-2 after cell transfer^[79-82]^. Due to its relatively high cost, it is also important to stratify patients who may clinically benefit from ACT. In this regard, reports have shown how the tumor mutational burden and the number of neoantigens may be good predictive criteria for ACT response^[83]^.

### CAR-T and TCR-engineered T cells

As explained above, adoptive transfer of autologous TILs has some limitations such as the low presence of specific antitumor T cells and the time needed to isolate and expand these cells from patients. For this reason, engineered T cells strategies have been developed, namely CAR-T cells and TCR-gene transfer among them. CAR-T cells are lymphocytes genetically engineered to express a chimeric receptor made by a single-chain variable fragment (scFv) of an antibody linked to an intracellular signaling domain by a spacer region and a transmembrane domain. The TCR-gene transfer technique consists in isolating an antitumor TCR sequence and its transfection inside lymphocytes^[84]^. Lentiviruses^[85]^ and DNA or mRNA transposon systems^[86]^ are the most common methods to transduce genetic materials.

The ectodomain of CAR can recognize surface antigens in an MHC-independent way, covering approximately 1% of the proteome^[87]^. This limit can be overcome by using TCR-like antibody fragments that specifically recognize a haplotype-restricted peptide complexed with MHC-I molecules^[88]^. The spacer region derives from CD28, CD8a of Fc IgG molecules, which changes CAR flexibility and off-target activities. Transmembrane regions can derive from different proteins such as CD3, CD28, CD8a or CD4. Finally, the intracellular domain allows classifying CAR-T cells into three generations, namely first-generation CARs containing only the CD3z internal region, second-generation CARs containing CD28 or 4-1BB (CD137) intracellular tails, and third-generation CARs including both costimulatory domains. Inclusion of both costimulatory domains does not always produce better results than the second-generation CARs. Most clinical trials, in fact, use second-generation CARs. A fourth generation, also referred to as TRUCK T cells, are CAR-T cells genetically engineered to constitutively express cytokines or their receptors^[89]^.

T cells can also be made insensitive to immunosuppressive signals. Different clinical trials (NCT02065362, NCT00368082, NCT03089203 and NCT01955460) are investigating the effects of engineered T cells expressing a truncated transforming growth factor beta (TGF-β) receptor 2 acting in a dominant negative manner in the tetramer receptor. New generations of CARs also contain suicide genes, co-expressed with the CAR construct to improve treatment safety. When off-target activities begin, genes can be induced by drugs or antibodies that trigger CAR cell apoptosis. The most commonly used are inducible caspase 9 and herpes simplex virus thymidine kinase^[90]^.

Until now, the success of CAR-T cell therapies has been strictly related to hematological malignancies; Kymriah (tisagenlecleucel) and Yescarta (axicabtagene ciloleucel) are two CD19-targeting CAR treatments approved by the FDA^[91,92]^ and EMA^[93]^ to treat, respectively, pediatric relapsed or refractory acute lymphoblastic leukemia, and relapsed or refractory diffuse large B cell lymphoma. CAR-T cell therapies for solid malignancies are not yet as successful, with many ongoing preclinical studies and clinical trials. Solid tumors are more difficult to target than hematological ones due to differences in the tumor-specific antigen burden and the presence of multiple physical barriers, such as the aberrant tumor vasculature, the extracellular matrix composition and TME suppressive elements^[94]^. Strategies are in place to overcome all these obstacles to effector functions, such as engineering of CAR-T cells to constitutively express catalase^[95]^ or hypoxia inducible factor 1 alpha (HIF1a)^[96]^, combination of CAR-T cell therapy with checkpoint inhibitors or, preferably, CAR-T cells co-engineered to secrete antibodies such as anti-PD-L1^[97]^ or anti-PD-1^[98]^ antibodies. Moreover, evidence from leukemia^[99]^ and melanoma patients^[100]^ treated with CARs shows how lymphodepletion before infusion with CAR-T cells increases the expansion, persistence and activity of these engineered cells. The standard regimen includes cyclophosphamide and fludarabine alone or combined^[101]^. Regarding PDAC, different antigens have been explored as targets for CAR-T and TCR-gene transfer strategies, namely mesothelin, HER2, altered glycosylated protein MUC-1, differentiation antigens, CEA, prostate stem cell antigen and CD24.

#### MSLN

MSLN is a cell surface protein expressed in the pleura, pericardium and peritoneum; it is also minimally expressed on epithelial cells of the tonsils, trachea, ovaries and testes^[102]^. Discovered in 2014, the *MSLN* gene encodes a pre-pro-protein proteolytically processed by furin protease to release two fragments, namely megakaryocyte potentiating factor, a cytokine capable of stimulating megakaryocyte colony formation inside the bone marrow, and MSLN, a glycosylphosphatidylinositol (GPI)-anchored cell surface protein that is important for cell adhesion. A soluble form of MSLN, known as soluble MSLN-related protein has been found in the serum of patients with solid tumors^[102]^. MSLN overexpression has been discovered in different tumors, including pancreatic cancer, in which almost 85% of cells express this protein^[103]^, and of course up to 90% of mesothelioma cells are positive for MSLN^[104]^. MSLN contributes to cell proliferation and resistance to apoptosis^[105]^, and by activating matrix metalloproteases 9 (MMP-9)^[104]^ and MMP-7^[106]^, it can induce tumor cell migration and invasion. Immunotherapy strategies targeting MSLN are under investigation in various tumor subsets, such as mesothelioma and lung and breast cancer^[107]^. A 2009 preclinical study evaluated the use of engineered TCR against MSLN to treat PDAC^[108]^. The authors isolated T cell clones specific for different epitopes of MSLN in both MSLN^-/-^ and WT mice; both mouse groups generated T cells against the MSLN fragment (406-414). T lymphocytes transfected with the highest affinity MSLN^-/-^-TCR (TCR1045) were able to bind MHC on target cells, independently from CD8 or CD4, displaying a higher lytic activity against PDAC MHC-I^+^ tumor cells compared to the highest affinity TCR from WT mice. At 8 days after the infusion of TCR1045-expressing T cells, which preferentially infiltrated the pancreatic mass, the authors observed increased tumor cell apoptosis. This was, however, a transient antitumor response, as T cell number decreased 18-fold in PDAC between 8 and 28 days after transfusion and those remaining, progressively upregulated the inhibitory receptors PD1, Tim3, Lag3 and 2B4^[108]^. In a randomized, blinded, placebo-controlled experiment, the authors confirmed how serial T cell infusions were able to enhance mouse survival and reduce metastatic disease and malignant ascites^[108]^. Concerning the use of CAR-T against MSLN in human pancreatic cancer, a phase I clinical trial was completed (NCT01897415) in March 2017^[109]^ [Table 1]. In this study, autologous T cells were transfected with RNA to transiently express the anti-MSLN CAR construct. Cells were injected intravenously three times per week for 3 weeks. Following RECIST 1.1 criteria, the authors observed using PET stable disease in two patients and a stabilization of metabolically active volume and maximum standardized uptake value (SUVmax) in three patients. At 2 months after the final injections, the presence of IgG antibodies was evaluated against an array of 9000 proteins; all patients generated a response against immune-related proteins, such as BCMA, PD-1 and PD-L1, but also against oxidative stress-related proteins^[109]^. Three other clinical trials are currently ongoing, including an interventional clinical trial at Nanjing Medical University (NCT03638193) started in August 2018. PDAC patients will be treated with CAR-T-meso lymphocytes 3 days after lymphodepletion with cyclophosphamide, to assess adverse events as the primary outcome and general clinical response as the secondary outcome. Patients’ own T cells will be isolated and lentivirally transduced to express a second-generation 4-1BB CAR construct containing a partial murine single-chain variable fragment, which is a feature that could easily lead to antibody-mediated elimination^[110]^. For this reason, a single arm phase I clinical trial started by Wenzhou Medical University in April 2018 (NCT03497819) is evaluating the combination of this second-generation 4-1BB CART-meso therapy with or without CAR-T cells directed against the CD19 biomarker of B lymphocytes. The novelty of this study is the comparison between two modalities of administration, i.e., 3 days after cyclophosphamide treatment, CAR-T cells will be injected either intravenously or via the pancreatic artery. A third observational trial (NCT04203459) started in October 2019 at Harbin Medical University for evaluating how gut microbiota can modify the proliferation, migration and functions of MSLN CAR-T cells.

#### Her2 and CD24

Her2 is a member of the epidermal growth factor receptor (EGFR) family. Her2 can heterodimerize with other members of the family, mediating cell growth and proliferation by activation of the MAPK and PI3K pathways. Known to be hugely amplified in breast cancer, Her2 is also overexpressed in about 60% of pancreatic cancers, correlating with a higher metastatic disease and worse outcome in resected patients^[111]^. CD24 is a GPI-sialoglycoprotein anchored to the cell surface, and it is essential for the growth and differentiation of granulocytes and B cells. In the tumor context, CD24, together with CD44 and CD133, identifies cancer stem cells^[112]^. Both antigens have been targeted by a CAR-T cell approach. Maliar *et al*.^[113]^ used a second-generation CD28 CAR-T against Her2 in a PDAC xenograft mouse model in which outcome was strictly related to Her2 expression on the tumor cell surface. In a second attempt, CD28 CAR-T cells targeting CD24 were injected after a preconditioning sub-lethal irradiation. CD24-specific CAR-T cells slowed xenograft growth and prolonged mouse survival. Treatments were also effective against high tumor burden and multiple metastases^[113]^. Unfortunately, a metastatic colon cancer patient treated with a third-generation CAR targeting Her2 died because of respiratory failure due to the recognition of Her2 in the lung epithelium^[114]^. The strong potency of CARs reduces the timeframe, after which antigens expressed on healthy tissues are targeted, increasing off-target toxicity. To extend this timeframe, Raj *et al.*^[115]^ developed a switchable CAR-T. The “switch” consisted in combining an anti-Her2 Fab tagged with a peptide neoepitope (PNE) and T cells engineered with a second-generation 4-1BB CAR directed against the PNE tag. In this way, CARs can be “switched on” only when the Fab binds Her2, acting as a bridge between target and effector. *In vitro* experiments confirmed how switchable CAR-T cells were able to lyse both patient-derived tumor cells and cancer stem cells^[115]^. Moreover, the authors demonstrated how switchable CARs worked as well as conventional CARs against stage IV PDAC human cells orthotopically injected in mice^[115]^. Tumor cell growth was monitored with live imaging and after 17 days, when tumors were detectable, mice were enrolled for treatment. Treatment with inactive Fab was ineffective, but at day 10, mice administered with either switchable or conventional CAR-T cells were tumor- and metastasis-free^[115]^. Moreover, no signs of graft-versus-host disease (GvHD) were noticed in mice treated with the switchable form of CARs, while two mice treated with conventional CARs developed GvHD-like symptoms^[115]^.

A phase I clinical trial referred to as VISTA (NCT03740256) is aimed at investigating a concurrent treatment - the intratumoral injection of oncolytic adenovirus (CAdVEC) modified to express genes coding for immune system stimulators, followed by injection of Her2 CAR-modified adenovirus cytotoxic T cells (Her2-AdVST). This approach will be tested on patients with Her2^+^ solid tumors, including pancreatic cancer as well as bladder, lung, breast, gastric and colorectal cancers. CAdVEC will be administered intratumorally with ultrasound guided percutaneous injection, while AdVST will be intravenously injected.

#### CEA

CEA, discovered by Gold and Freedman in 1965^[116]^, is a cell surface protein with a GPI domain and is expressed from an early fetal period (9-14 weeks) throughout life. Detected at very low levels in healthy individuals, it is used as a tumor marker for colon, breast, liver, lung, stomach and pancreatic cancers, with 75% of the last expressing CEA. CEA can act as a homophilic or heterophilic adhesion tool in cancer cells^[117]^. CEA was previously exploited as a target in other immunotherapeutic strategies such as vaccines, while the CAR approach for this antigen began in 2012: Chmielewski *et al.*^[118]^ used second-generation CD28 CAR-T cells containing the murine antibody SCA431scFv directed against an epitope of the membrane proximal CEA domain. This strategy avoided dose-limiting autoimmune colitis and pneumonia observed in a pilot study for metastatic colorectal cancer^[119]^. Panc02 pancreatic carcinoma cells were injected into the pancreas of immune-competent transgenic mice, expressing CEA in the gastrointestinal and pulmonary tracts^[118]^. Without any lymphodepletion preconditioning, 10 days after tumor transplantation, autologous T cells were inoculated via the tail vein in a single dose^[118]^. A specific antitumor activity was generated without any kind of autoimmune pathology^[118]^ observed in the trial^[119]^. This was achieved due to the low-affinity CAR-T cells used in this preclinical model. CAR-T cells at cancer lesions were characterized by an exhausted phenotype (CD62L^-^, CD127^-^ and CD279^-^) because of the low concentration of cytokines in the TME responsible for T cell activation, proliferation and degranulation^[120]^. To bypass these limitations, Chi *et al*.^[121]^ combined the administration of an anti-CEA second-generation 4-1BB CAR with the intravenous injection of rhIL-12 to increase interferon gamma (IFN-γ) secretion and cytotoxicity of both natural killer (NK) and T cells. CAR-T cells co-cultured *in vitro* with CEA^+^ pancreatic cell lines, and treated with rhIL-12, showed a higher expression of cell surface proteins CD69 and CD25, a higher production of IL-2 and IFN-γ, greater cell proliferation and stronger cytotoxic activity than CAR-T cells not treated with the cytokine. The same CEA^+^ pancreatic cell lines were intravenously injected into mice, followed on day 7 by intravenous injection of anti-CEA CAR-T cells with or without rhIL-12 treatment. Adding the cytokine rhIL-12 enhanced the anti-metastatic activity of CAR-T cells, together with a higher CD8^+^/CD4^+^ circulating cell ratio and production of the serum cytokines: IFN-γ, IL-2 and tumor necrosis factor alpha (TNF-α) compared to mice not treated with the cytokine^[121]^. Similarly, Chmielewski and Abken^[122]^ exploited second-generation CD28 anti-CEA CAR-T cells transduced with an inducible IL-18 cassette under the control of IL-2 promoter. In this way, engineered T cells were able to express IL-18 only after antigen recognition and dephosphorylation of nuclear factor of activated T cells, an inducer of IL-2 expression. IL-18 was found to be responsible for the effector T cell signature acting on Tbet and FoxO1^[122]^. Double-engineered CARs were able to prolong the survival of mice with long-term established orthotopic disease, together with a reduction of M2 macrophages and Tregs. Zhang *et al.*^[123]^ also developed dual second-generation 4-1BB CAR-T cells targeting both MSLN and CEA. CD3-mediated activation was provided by the construct that recognized CEA, while 4-1BB mediated the co-stimulation signal by the construct that recognized MSLN. These dual CAR-T cells showed both *in vitro* and *in vivo* activities comparable to conventional CAR-T cells^[123]^. The off-target toxicities were strongly reduced, but dual CARs were only able to exert antitumor activity when both antigens were expressed on the tumor cell surface^[107,123]^. Two clinical trials are currently evaluating the effects of anti-CEA CAR: Roger Williams Medical Center started an open-label, single-arm phase 1b clinical trial (NCT03818165) in January 2019 for liver metastatic PDAC. This trial will involve infusion of second-generation anti-CEA CAR-T cells together with IL-2, and is expected to be completed in February 2022. Still concerning liver metastatic PDAC, Sorrento Therapeutics in California started an open-label phase 2b clinical trial (NCT04037241) in July 2019. Patients with progressive disease after first-line treatment with FOLFIRINOX or gemcitabine will receive second-generation anti-CEA CAR-T cells alone or combined with gemcitabine or nab-paclitaxel.

#### MUC-1

MUC-1 is a transmembrane protein composed of a tandem repeat (TR) containing an external domain, bound to the membrane through non-covalent interactions with the MUC-1-C domain, made of a short extracellular domain and transmembrane and cytoplasmic domains. In tumors, MUC-1 shows a different *O*-glycosylation of the serine and threonine residues in the TR domains^[124]^. The most common aberrant glycosylated form can bind the sialic acid-binding protein Siglec-9 expressed by monocytes and macrophages; after this interaction, tumor-associated macrophages (TAMs) increase expression of PD-L1^[125]^. The aberrant form of MUC-1 is expressed in more than 80% of PDAC cases, which makes MUC-1 a good target for cancer immunotherapy^[126]^. Second-generation 4-1BB CAR-T cells exploiting the domains of the 5E5 antibody, which specifically recognizes the aberrantly glycosylated MUC-1, were used to treat disseminated tumor xenografts of intraperitoneally injected Hs766T pancreatic cells. All mice treated with anti-MUC-1 CAR-T cells survived, while the survival of mice treated with untransduced CAR-T cells or unrelated CD19 CAR-T cells was 40% and 33%, respectively^[125]^. CD28 anti-MUC-1 CAR exploiting the variable fragments of another tumor-specific antibody (TAB004) was used in a preclinical study published in *Cells* in 2019^[127]^. A study using a xenograft model with the MiaPaCa2 cell line demonstrated how anti-MUC-1 CAR-T cells were able to reduce tumor weight and metastasis^[127]^. From the *in vitro* assay, five genes were found to be upregulated in two non-responding human cell lines (CFPAC and HPAFII), namely *IDO-1*, *COX1* and *COX2, ADAR1* and *Gal-9*. Exploiting small molecule inhibitors and antibodies against these genes, investigators were able to restore sensitivity to CAR-T cell treatment *in vitro*^[127]^. No clinical trials are currently testing anti-MUC-1 CAR-T cells in PDAC patients.

#### Prostate stem cell antigen

Prostate stem cell antigen (PSCA) is a glycoprotein composed of 123 amino acids anchored to the cell surface membrane by GPI. PSCA is undetectable in the healthy pancreas but overexpressed in PDAC, as well as in prostatic, urinary and bladder tumors, where its functions remain unclear. The difference in PSCA expression between normal and tumor tissues is more pronounced than with MSLN. Second-generation CD28 and third-generation CARs were developed by Abate-Daga *et al.*^[128]^ and functionally compared to anti-MSLN CARs currently used in clinical trials. Anti-PSCA CARs induced larger amounts of IFN-γ than did anti-MSLN CARs when co-cultured with pancreatic cancer cell lines. Moreover, preclinical experiments demonstrated that second-generation CARs, but not third-generation CARs, were able to strongly reduce subcutaneous growth of HPAC pancreatic cells, with tumor rejection in two out of five mice^[128]^. To overcome the immunosuppression in the TME, Mohammed *et al.*^[129]^ generated a first-generation of anti-PSCA CAR-T cells co-transduced with a chimeric switch receptor, in which the extracellular domain of IL-4R is linked to the immunostimulatory intracellular domain of IL-7R, to exploit the IL-4 inhibitory signal in the activation of the IL-7 pathway. The double transduction was able to increase the destruction of CAPAN-1 cells subcutaneously engrafted in NOD/SCID/IL-2Rg^null^ (NSG) mice^[129]^. Bellicum Pharmaceuticals in Houston started a phase I clinical trial (NCT02744287) in April 2016 to evaluate the efficacy and safety of BPX-601, an anti-PSCA CAR that also contains a rimiducid-inducible signaling domain able to enhance CAR-T cell proliferation and activation. The estimated primary completion date is set for February 2022.

#### Other alternative antigens

Some “unconventional” antigens were tested as targets for CAR-T cell therapies, including CD47, B7-H3 and integrin αvβ6. CD47 is a cell surface glycoprotein belonging to the immunoglobulin superfamily and represents a “don’t eat me signal”, able to inhibit phagocytosis by binding to signaling regulatory protein α (SIRP-α). CD47 is overexpressed in leukemia and lymphoma, and in ovarian cancer, small cell lung cancer, glioma, glioblastoma and pancreatic cancer as well, where it represents a negative prognostic factor^[130]^. A second-generation CD28 anti-CD47 CAR-based treatment was able to significantly decrease the volume and weight of tumors developed in mice subcutaneously injected with BxPC3 pancreatic cells^[130]^. B7-H3, instead, is a member of the B7 costimulatory or co-inhibitory family delivering an immunosuppressive signal. With a limited expression in normal tissue, B7-H3 is expressed by the tumor-associated vasculature and fibroblasts, and correlates with poor prognosis and outcome^[131]^. Two second-generation, CD28 or 4-1BB, anti-B7-H3 CARs were able to completely reject PANC1 orthotopic tumors, with all mice remaining tumor-free for more than 80 days^[131]^. Finally, αvβ6 is an integrin found overexpressed in pancreatic, head and neck, skin, lung, stomach, colon and breast cancers^[132]^. This integrin activates TGF-β, promoting the migration and activation of MMPs and the epithelial-to-mesenchymal transition. A second-generation CD28 anti-αvβ6 CAR was used to target BxPC3 and PANC0403 cell lines intraperitoneally inoculated into severe combined immunodeficient beige mice. These CAR-T cells were able to cause the tumor regression growth with regard to both cell lines^[132]^.

These data support this tailored approach as a powerful tool for targeting solid tumors, including PDAC.

## Monoclonal antibodies

Passive immunotherapies, mainly represented by monoclonal antibodies, are efficacious in many types of cancers, and the first used in clinical practice were EGFR- or VEGF-antibodies in colorectal cancer, and trastuzumab in Her-2-positive breast cancer. Her-2 is also widely expressed on PDAC cells, and trastuzumab therapies employ antibody-dependent cellular cytotoxicity (ADCC) mediated by NK cells for destruction of tumor cells. In 2018, Iwakiri’s group reported that combined therapy of trastuzumab with anti-CD137 (4-1BB) could improve NK-mediated ADCC of human PDAC cell lines *in vitro*^[133]^. This was related to an increased expression of CD137 on NK cells in response to trastuzumab, associated with the Fcg-RIIIA-VV/VF polymorphism^[133]^. Other targeted molecules have, so far, been unsuccessful in pancreatic cancer, such as cetuximab and bevacizumab^[134]^.

### Myeloid targeting approaches

A promising molecule combining adaptive and innate immunity is CD40, a member of the TNF receptor family^[135]^. CD40 is expressed on monocytes and DCs and also B cells, platelets and non-hematopoietic cells and tumor cells^[136,137]^. The ligand of CD40 (CD40L or CD154) is expressed following activation by CD4^+^ and CD8^+^ T cells, B cells, NK cells and macrophages^[138]^. The significance of CD40 in tumor immunology was highlighted by a series of pioneering articles that showed that administering an agonistic antibody induced protective T cell immunity in murine cancer models of T-cell lymphoma and renal carcinoma^[139,140]^. CD40 agonists have been exploited to ameliorate the three immune cell cycle steps: tumor killing, T-cell immunity induction and immunosurveillance activation^[141]^ [Figure 1]. The most widely used agonist is the fully human CD40-specific monoclonal antibody called CP-870,893. Even if CD40 targeting is effective in activating the immune system, its clinical activity is fairly limited. Therefore, it is clear that combination with other treatments is pivotal for effective translation of the CD40 agonist. Some preclinical modeling has aimed to combine CD40 antibodies with TLR agonists, showing how the combined therapy achieved 10- to 20-fold greater responses than the agonists alone by synergizing to stimulate CD8^+^ T cell responses, as demonstrated by increased lytic activity and IFN production^[142]^. Alternatively, CD40 antibodies have been combined with IL-2 immunotherapy as a strategy to induce tumor-specific T cell immunity in tumor models of renal cell adenocarcinoma or Lewis lung carcinoma. CD40 stimulation and IL-2 synergize to induce complete regression of metastatic tumors due to potentiation of T cell survival and CD40 expression of DCs, stimulating an immunomodulatory cascade^[143]^.

To understand the mechanisms of action of the anti-CD40 antibody, many studies have used a genetically engineered mouse model of pancreatic cancer^[5]^. Using an agonist rat anti-mouse mAb as a prototype for CP-870,893, Beatty *et al.*^[138]^ demonstrated a T cell-dependent and -independent mechanism induced by the agonist. In orthotopic PDAC models, anti-CD40 treatment was associated with an increased control of tumor growth. Notably, in a second set of experiments, mice spontaneously developing pancreatic tumors treated with the anti-CD40 mAb FGK45 and gemcitabine showed a major tumor regression compared to gemcitabine alone, supporting that observed in the previous model^[144]^. In addition, the same rate of tumor regression was obtained with anti-CD40 mAb alone as that produced by combination with gemcitabine. Previous studies have suggested that CD40-activated macrophages can inhibit tumor growth due to IFN-γ production, indicative of a classically-activated macrophage phenotype^[144]^. Administration of anti-CD40 in PDAC-bearing mice did not produce a significant change in the number of TAMs within the tumor, but a transitory change in macrophage activation (i.e., CD86 and MHC class II expression) was seen within 24-48 h of treatment^[138]^. Moreover, macrophages isolated from the pancreas of tumor-bearing animals treated *in vivo* with anti-CD40 mAb were capable of lysing tumor cells *in vitro*^[138]^. Furthermore, treatment of PDAC-bearing mice with clodronate abrogated anti-CD40-mediated tumor regression^[138]^. These findings reinforce the idea that CD40 immune therapy may be dependent on macrophages, and that the CD40 pathway can be harnessed to restore tumor immunosurveillance by targeting tumor-infiltrating macrophages. This particular study did not investigate the role of B cells and DCs, although both cells can be activated by the CD40 agonist or anti-CD40 mAb^[145]^. Indeed, Schultze *et al.*^[146]^ discovered that fibroblasts expressing CD40L could be used in association with IL-4 to generate large numbers of human B cells that act as APCs to generate antitumor CD8^+^ T cells. More recently, CD40L-expressing fibroblasts have been replaced with soluble CD40L^[147]^.

Regarding DCs, monocyte-derived DCs treated with CP-870,893 exhibited a mature phenotype, with upregulation of CD80, CD83, CD86 and HLA-DR, and increased Mip1α and IL-12 secretion, indicating that CP-870,893 binds and activates DCs *in vitro*^[148]^. In PDAC mouse models, classic DCs are a rare population in the TME expressing CD40^[149]^. Upon activation of CD40 on DCs, many other agonistic pathways such as GITR, OX40 and 41BB are activated. As DCs have an enhanced capacity to take-up antigens when immature^[150]^, chemotherapy followed by CD40 activation but not vice versa, elicited effective T-cell dependent immunity in tumor-bearing mice compared with anti-CD40 alone^[151,152]^. In subcutaneously implanted PDAC tumors, gemcitabine followed by agonistic CD40 is effective in inducing IFN-γ and TNF-α secreting T cells, activated DCs, and limiting M2 macrophages and Tregs^[153]^. However, the addition of nab-paclitaxel to gemcitabine, which is more effective in patients than gemcitabine alone, synergizes to trigger tumor regression and improve survival in spontaneous PDAC mouse models^[152]^. Notably, chemotherapy plus anti-CD40 failed both in CD40^-/-^ and Batf3^-/-^ mice (which lacked cross-presenting DCs), suggesting a critical role of DCs^[152]^. Given the preclinical success, CD40-targeting agents have progressed to the clinical phases. In collaboration with Pfizer Corporation, a two-center trial at the University of Pennsylvania (PI, Peter O’Dwyer) and Indiana University (PI, Elena Chiorean) examined the combination of gemcitabine and CP-870,893 in untreated patients with advanced pancreatic adenocarcinoma (NCT00711191) [Table 1]. Though gemcitabine alone has, historically, produced a partial response of approximately 5% or less in the chosen settings, 4 patients out of 21 were reported to have a partial response and 11 patients had stable disease as the best response^[154]^. A follow-up clinical study of CP-870,893 with gemcitabine is now ongoing for patients with resectable PDA at the University of Pennsylvania (NCT01456585)^[138,155]^.

Granulocyte-MDSC (Gr-MDSC) are another interesting target; these cells are abundant in the TME and their increase correlates with PDAC progression^[156]^. Stromnes *et al.*^[156]^ depleted these populations using a mAb targeting Ly6G^+^ cells in a spontaneous PDAC mouse model. Treatment with this antibody achieved effective intratumoral Gr-MDSC depletion for over 2 weeks accompanied by an accumulation of CD8^+^ T cells. Although there were no observed differences in tumor growth, Gr-MDSC depletion decreased ECM deposition and enhanced caspase-3 cleavage in cancer cells.

Colony-stimulating factor-1 (CSF-1) acts as a monocyte attractant and is one of the main growth factors for monocyte-lineage cells. Moreover, CSF-1 induces polarization of macrophages into a tumor-promoting phenotype^[157,158]^. CSF-1 tyrosine kinase receptor (CSF-1R), has been the focus of attention by scientists and companies who have developed a number of small molecules and antagonists to block its action^[159]^. In PDAC preclinical mouse models, blocking the CSF-1/CSF-1R pathway was achieved through either CSF-1-neutralizing antibodies or CSF-1R inhibitors^[160]^. Abrogation of the CSF-1 axis was sufficient to redirect TAMs towards an M1 pro-inflammatory phenotype in metastatic lesions^[160]^. Zhu *et al.*^[161]^ showed that treatment with an anti-CSF-1 mAb resulted in enhanced PD-1 and cytotoxic T-lymphocyte ntigen 4 (CTLA-4) expression in TILs. Therefore, they suggested combining inhibition of the CSF-1/CSF-1R axis with ICI therapy, particularly for single-agent resistant tumors. In preclinical PDAC models, this combined therapy led to regression of established orthotopic tumors^[161]^. Similarly, Mitchem *et al.*^[162]^ showed how the CSF-1R inhibitor reduced the amount of CD11b^+^ Ly6G^-^ Ly6C^low^ MHC-II^high^ F480^+^ macrophages that infiltrated into murine PDAC tumors. Moreover, combination treatment with the CSF-1R inhibitor and gemcitabine restored antitumor activity to TILs, demonstrated as increased levels of IFN-γ, IFN-β1, granzyme B, perforin, and IL-12 p35 and decreased expression of TGF-β and Arg-1 mRNA in tumors^[162]^. To evaluate the safety and clinical activity of a combined treatment, a recruiting early phase I trial (NCT03153410) is evaluating the combination of cyclophosphamide, pembrolizumab, GVAX and IMC-CS4 (LY3022855) (an anti-CSF-1R mAb) in patients with borderline resectable pancreatic cancer.

### Immune checkpoints

In physiological conditions, the activation of T cells is counterbalanced by inducing inhibitory stimuli to maintain immune homeostasis and to limit tissue damage and autoimmune reactions. Signals and regulators involved in these kinds of inhibitory responses are known as immune checkpoints (IC). Among the molecules responsible for the negative regulatory processes, the most studied and characterized are CTLA-4 and PD-1. CTLA-4 is mainly expressed on the surface of Tregs, naïve T cells and effector T cells. CTLA-4 competes with the co-stimulatory receptor CD28 in binding CD80 (also known as B7-1) and CD86 (also known as B7-2) molecules, to modulate the strength of the activated T cell responses or to enhance Treg-mediated immunosuppression^[163]^. PD-1, expressed on the surface of T cells, B cells and NK cells, interacts with PD-1 ligand 1 (B7-H1) and ligand 2 (B7-DC), influencing both central and peripheral tolerance mechanisms^[164-166]^. Activating IC pathways combined with the recruitment of suppressive cell populations, is a typical immune-escape mechanism adopted by different types of cancers^[167,168]^. PDAC is considered an “immunologically cold” tumor because of the high infiltration of Tregs and MDSCs and the consequent impairment of CD4^+^ and CD8^+^ cell-mediated antitumor responses^[169,170]^. We have previously demonstrated that the tumor area is infiltrated by Tregs and Th17 cells that frustrate effector T cells much more than the normal surrounding mucosa^[171]^. However, the presence of CD4^+^ and CD8^+^ T cells in the tumor has been demonstrated to correlate with better prognosis^[172]^. A recent retrospective study of 453 PDAC samples investigated PD-L1 mRNA expression levels and revealed an upregulation in 19% of the samples^[173]^. Rahn and colleagues suggest that in pancreatic cancer, PD-L1 is mainly expressed on the surface of stromal and MDSC cells^[174]^.

Taking these premises into account, the use of ICIs to disrupt Treg-mediated immunosuppression and to block the inhibitory pathways on effector T cells, seems to be potentially effective in PDAC treatment [Figure 1].

#### ICI monotherapy

The mechanism of action of ICIs is to stimulate immune-mediated recognition and elimination of the tumor. Ipilimumab is the first anti-CTLA-4 monoclonal antibody approved for treating metastatic melanoma patients^[175]^. Although the use of anti-CTLA-4 antibody in PDAC mouse models suggests potential benefits such as the increase of CD4^+^ cell recruitment^[176]^, no effects were observed in tumor growth reduction or overall survival^[149]^. These results were also confirmed by a phase II trial (NCT00112580) in which the single agent ipilimumab was tested for the treatment of locally advanced or metastatic pancreatic adenocarcinoma [Table 1]. Only one out of 27 subjects showed a significantly delayed response, suggesting that ipilimumab monotherapy is ineffective for treating advanced PDAC^[177]^. Even anti-PD-1 mAbs (such as pembrolizumab and nivolumab) and anti-PD-L1 mAbs (such as ateolizumab, durvalumab and avelumab) have been approved by the FDA for treating different solid tumors^[178]^. In mice subcutaneously injected with PAN02 (PDAC cell line), anti-PD-L1 treatment significantly reduced tumor growth and increased the number of CD8^+^ cells producing IFN-γ, granzymes and perforins^[179]^. Unfortunately, opposite results were observed in a genetically engineered PDAC mouse model, in which anti-PD-L1 treatment alone was ineffective^[149]^. Moreover, as described previously for anti-CTLA-4, anti-PD-L1 monotherapy failed in the clinical approach. All 14 patients with advanced pancreatic cancer who were enrolled in a phase I clinical trial (NCT00729664) showed no responses to treatment with BMS-936559 (an anti-PD-L1 antibody)^[180]^ [Table 1]. Despite this, the anti-PD-1 antibody seemed to be effective in a small PDAC patient cohort with mismatch repair (MMR) deficiency. In a study conducted by Le *et al.*^[181]^ (NCT01876511), eight PDAC patients were also enrolled for evaluating PD-1 blockade efficacy in patients with advanced MMR-deficiency in different kinds of cancers [Table 1]. Treatment with pembrolizumab was able to induce a complete response in two patients, a partial response in three patients and stabilization of the disease in one patient (the remaining two patients could not be evaluated)^[181]^. The high mutation rate observed in MMR-deficient tumors, due to the failure of the DNA repair mechanisms, increases their immunogenicity and makes them suitable for checkpoint inhibitor treatments. Unfortunately, this is the case for only 1% of PDAC patients^[182]^.

One of the reasons for the failure of ICIs in treating pancreatic cancer may be the highly immuno-suppressive TME, which makes it comparable to an immune-privileged site^[183]^. To overcome these adverse conditions, ICIs were tested in combination with treatments that have been shown to affect steps of the cancer immunity cycle that are deficient in PDAC.

#### ICIs and chemotherapy

To date, the gold standard of care for PDAC treatment is based on the use of gemcitabine (with or without nab-paclitaxel) and FOLFIRINOX^[184]^. Chemotherapeutic agents not only have a direct effect on cancer cells (by impairing cell division or increasing the DNA-mutation rate and therefore their death), but can also boost the immune system response by inducing so-called “immunogenic cell death” (ICD). Briefly, upon chemotherapy, cancer cells undergo necrosis thereby releasing molecules that act as danger signals which, in turn, activate the innate immune system. Furthermore, it has been shown that some chemotherapeutic agents can increase MHC-I expression on the surface of the cancer cells and promote maturation of DCs. Overall, chemotherapy improves the immunogenicity and antigenicity of the tumor^[185]^ by enhancing potential benefits from the coupling with ICIs. Nomi and colleagues demonstrated that combining gemcitabine with PD-L1 blockade in PDAC resulted in a complete antitumor response in a subcutaneous injection PDAC mouse model^[179]^. In another study, Winograd *et al.*^[149]^ by using a combination of gemcitabine/nab-paclitaxel plus anti-PD-1 and anti-CD40 mAbs observed regression of the tumor mass and an improved median overall survival of tumor-bearing mice. Many clinical trials evaluate the potential of combining ICIs with chemotherapy in PDAC patients^[186]^. Gemcitabine combined with the anti-CTLA-4 antibody has been evaluated in two different phase Ib trials. Respectively, 34 and 16 patients were treated with gemcitabine with or without tremelimumab^[187]^ and ipilimumab^[188]^. There was a partial response in 6% of patients, and 21% of patients achieved stable disease with an overall survival of 7.4 months in the tremelimumab trial (NCT00556023). In the ipilimumab trial (NCT01473940), 14% of patients achieved an objective response rate and 33% of patients achieved stable disease with an overall survival of 6.9 months^[187,188]^ [Table 1]. Blocking PD-1 through pembrolizumab, used in combination with gemcitabine/nab-paclitaxel, was evaluated in 17 patients with metastatic PDAC (5 out of 17 had received prior chemotherapy) in a phase Ib/II study (NCT02331251) [Table 1]. For the chemotherapy naïve patients, the overall survival was 15 months with a partial response and stable disease in 25% and 67% of patients, respectively. Interestingly, no patients had progressive disease^[186,189]^. A combination of nivolumab/nab-paclitaxel with or without gemcitabine was evaluated in 42 naïve treatment patients with advanced pancreatic cancer (NCT02309177) [Table 1]. The overall survival was 9.9 months and 2% of patients showed a complete response, 16% a partial response and 46% stable disease^[190]^. Many different trials with ipilimumab, avelumab, pembrolizumab, nivolumab and durvalumab are ongoing, either given alone or combined with chemotherapy and other biological drugs^[186]^.

#### ICIs and radiotherapy

As previously described for chemotherapy, radiotherapy (RT) can also induce ICD of cancer cells and enhance the antitumor immune response^[191]^. In particular, it has been documented that RT can induce the so-called “abscopal effect”, by which treatment of a primary tumor can induce the immune-mediated shrinkage of secondary lesions^[192]^. This mechanism occurs for CD8^+^ T cells that migrate to secondary lesions after activation in the RT-treated primary tumor, where they recognize the same antigen by which they were primed^[193]^. Therefore, even if RT is not the gold standard treatment in PDAC, some studies have shown the benefits that derive from combining RT with ICIs^[184]^. Azad and colleagues demonstrated that RT and gemcitabine can induce overexpression of PD-L1 in syngeneic PDAC tumor allografts. Considering these findings, they decided to investigate RT in combination with anti-PD-L1 and/or gemcitabine. PD-L1 blockade in association with RT significantly delayed tumor growth, which was enhanced by the addition of gemcitabine^[194]^. Similarly, Victor *et al.*^[195]^, demonstrated the efficacy of combined treatment with RT plus anti-PD-1 and anti-CTLA-4 in a subcutaneous PDAC model. Furthermore, more positive results were obtained in tumor-bearing mice treated with a combination of RT, anti-CD40 and anti-CTLA-4/PD-1^[196]^. To date, some clinical trials are ongoing to better evaluate different combinations of ICIs and RT in pancreatic cancer; for example, a combination of nivolumab, ipilimumab and RT are used in the trials NCT03104439 and NCT02866383; and tremelimumab and/or MEDI4736 (anti-PD-L1) in combination with RT are used in the trials NCT02311361 and NCT02639026.

#### ICIs and vaccination

As previously mentioned, one of the most used cancer vaccines in PDAC is GVAX. In a phase II study, its ability to induce cancer-specific CD8^+^ T cell expansion and formation of tertiary lymphoid organs was reported, suggesting the potential of GVAX to reverse PDAC from a “non-immunogenic” into an “immunogenic” state^[19,21,197]^. According to these findings, it was suggested that a combination based on vaccination and ICI could induce an effective anticancer response. In a phase I study, ipilimumab alone, or combined with GVAX was evaluated in 30 previously treated advanced PDAC patients [Table 1]. The median OS of patients receiving the combined treatment was 5.7 months compared to 3.6 months in patients receiving ipilimumab alone. In patients with an OS longer than 4.3 months, there was an increase in the peak of mesothelin-specific T cells, and an expansion of the T cell repertoire^[198]^. Soares and colleagues^[199]^ observed that GVAX significantly upregulated PD-L1, and that combining GVAX with anti-PD-1 mAb improved mouse survival compared to monotherapy. This result was also supported by an increase in recruitment of tumor-specific effector CD8^+^ T cells producing IFN-γ^[199]^. Similarly to that observed in preclinical experiments, Lutz *et al.*^[200]^ reported that 33 out of 39 resected tumor tissues of GVAX-vaccinated patients were characterized by the presence of intratumoral tertiary lymphoid aggregates, together with an upregulation of the PD-1/PD-L1 pathway. Hence, many clinical trials are evaluating the most efficacious combination therapies, for example, GVAX combined with nivolumab alone (NCT02243271, NCT02451982, NCT03161379, NCT03767582) or combined with either ipilimumab (NCT03190265) or pembrolizumab (NCT02648282)^[201]^.

#### ICIs and adoptive T cell therapy

The lymphocytes used for adoptive T cell therapy (both autologous and allogenic T cells) face the same problem deriving from TME immunosuppression described above. Therefore, in this case as well, combined treatment with ICIs could increase ACT efficacy. ACT flop is often connected to enhanced expression of the T cell inhibitory marker, namely PD-1^[202,203]^. Ironically, recognition of tumor cells by transferred T cells leads to T cell activation and consequently upregulation of PD-1 on their surface; similarly, tumor cells enhance PD-L1 expression in response to T cells. This ultimately leads to abrogation of T cell activity and thus ACT failure^[204]^. Evidence that this anergic state could be reverted when the PD-1/PD-L1 axis is blocked has emerged from several phase III clinical trials in non-small cell lung cancer and melanoma^[205,206]^. On the basis of these reports, a similar approach has also been assessed in PDAC mouse models^[207]^, testing an approach to transform the PD-1/PD-L1 immunosuppressive axis for stimulation of TILs through transfection of a fusion protein, consisting of the extracellular PD-1 domain fused with the intracellular T-cell-activating CD28 protein, in the transferred T cells. It was shown that murine CD4^+^ T cells expressing the PD-1-CD28 fusion protein can overcome PD-L1-induced T cell inhibition.

Furthermore, John and coworkers described a significant improvement in the growth inhibition of two different Her2^+^ tumors treated with anti-Her2 T cells combined with an anti-PD-1 mAb in tumor-bearing mice with subcutaneous injection of tumor cells. The therapeutic effects correlated with an increased function of anti-Her2 T cells and a significant decrease in tumor-infiltrating MDSCs^[208]^. Regarding pancreatic cancer, a phase II clinical trial is investigating the combination of ACT therapy and ICI in patients with metastatic PDAC. These patients will receive an infusion of autologous PBMC-derived T cells with IL-2 and pembrolizumab after chemotherapy (NCT01174121)^[209]^. In addition, novel studies are focused on autologous CAR-T cells engineered to recognize specific solid tumor antigens and to simultaneously express anti-PD-1 and anti-CTLA-4 antibodies in non-hematologic malignancies (NCT03182803, NCT03030001, NCT03179007). Moreover, a new strategy based on *PD-1* gene depletion by using CRISPR/Cas9 technology is being developed to engineer CAR-T cells and make them insensitive to checkpoint inhibitory stimuli^[210]^.

## The unique pancreatic cancer microenvironment

The PDAC TME is characterized by cells exerting both anti-tumor and pro-tumor functions such as endothelial cells, fibroblasts, lymphocytes and myeloid-derived cells. These populations are able to influence therapeutic responses and to sustain tumor progression by compromising the antitumor activities of effector cells. Therefore, PDAC TME is considered one of the most relevant causes of immunotherapy failure in the treatment of this disease. TILs may encounter a suppressive environment involving inhibitory cytokines, hypoxia, as well as a dense fibrotic stroma and abnormal blood vessels that act as physical barriers preventing them from functioning properly. Other negative regulators, as already discussed, are the immune checkpoints expressed by tumor cells and myeloid-derived cells, such as PD-L1 and CD80/CD86, respectively, which interact with PD-1 and CTLA-4 expressed on the T cell surface. These interactions generate negative signals leading to immune suppression^[157]^. In particular, Candido *et al.*^[211]^ confirmed that TAMs are the principal source of PD-L1 expression in the PDAC TME. TAMs can derive from the differentiation of monocytic-MDSCs (M-MDSCs), recruited by cancer-produced CCL-2 and CCL-5, and differented in macrophages inside the TME by stabilizing HIF1α in the tumor hypoxic condition. Furthermore, HIF1α binds to the PD-L1 promoter hypoxia-responsive element motifs, rendering those cells capable of suppressing T cell activation in an antigen-nonspecific manner^[212]^. In addition, macrophages can induce fibrosis, a key element of T cell exclusion from the TME^[160]^. TAMs with a M2-like polarization can directly and indirectly suppress CD8^+^ activity; by expressing TGF-β, they are able to repress levels of perforin and granzymes, and with the help of IL-10 they induce Tregs. These CD4^+^CD25^+^Foxp3^+^ T cells can derive from maturation in the thymus or by conversion of conventional T cells upon TCR stimulation together with TGF-β. Treg cells express CTLA-4 and predominantly consume IL-2 due to the high expression of CD25, depriving this cytokine of effector cells. Moreover, it has been demonstrated that Tregs are able to mediate an immunosuppressive mechanism through the co-expression of CD39 and CD73 molecules on their surface. In fact, CD39 converts ATP and ADP into cAMP that, in turn, is converted into adenosine by CD73. Adenosine binds to specific receptors on CD4 and CD8 T cells as well as NK cells, macrophages and DCs by inhibiting antitumor cell response^[213,214]^. In addition, Tregs can directly kill effector cells via perforin or granzyme release^[215]^.

A similar pro-tumor microenvironment renders pancreatic cells capable of evading immune surveillance, especially because of the impaired tumor antigen presentation by DCs, which are not “classically activated”^[216,217]^. The immunosuppressive state, responsible for the failure of most of immunotherapeutic approaches, can be directly sustained also by tumor cells. Fogar *et al.*^[218]^ showed how pancreatic cancer cells themselves release soluble mediators able to suppress the proliferation and migration of CD4^+^ T cells, expanding CD69^+^CD4^+^ T cells with an inhibitory role on conventional CD4^+^ in a contact-dependent way. Therefore, different from more immunogenic tumors such as renal cell carcinoma or melanoma, which attract tumor-infiltrating effector cells^[219]^, pancreatic cancer shows poor immunogenicity, which results from both dysfunctional antitumor response and surmounting immunosuppression.

Deeper genetic characterization of solid tumors has led to the identification of specific subtypes of the same histotype tumor that benefit from different treatments. Thanks to an integrated genomic analysis of 456 cases of pancreatic ductal adenocarcinoma, 4 different tumor subtypes were identified: squamous, pancreatic progenitor, immunogenic and aberrantly differentiated endocrine exocrine. In particular, the immunogenic subtype is associated with a significant immune infiltrate, B cell signaling pathways, antigen presentation, CD4^+^ T cell, CD8^+^ T cell and Toll-like receptor signaling pathways. Of note, this subgroup displayed upregulated CTLA-4 and PD-1 suppressive pathways, suggesting a higher potential response to the inhibition of immune checkpoints compared to the other subtypes. However, stratification based on tumor immunological background might identify those patients who will benefit from immunotherapy strategies in general^[220]^.

Indeed, immunotherapy responsiveness can be explained and predicted thanks to the combination of two independent elements: the “hot” or “cold” status, according to the range of inflammatory cell infiltration and IFN-γ signature, and the tumor mutation burden, which can lead to neoantigen expression. Melanoma and non-small cell lung cancer are hot tumors with a high tumor mutation burden, making them highly antigenic and immunogenic and thus potentially more sensitive to immunotherapy^[221]^. Unfortunately, PDAC, alongside a strong immunosuppression, has a low number of genomic mutations in protein coding regions. This small antigen mutational burden may be another reason for the difficulty of inducing an effective antitumor T cell response^[201]^.

## Conclusion

“Dark side” in the title can be considered a reference to the evil force in the famous Star Wars saga. Due to many of its intrinsic characteristics, including a well-established desmoplastic reaction and an immunosuppressive microenvironment, pancreatic cancer actually represents the dark side of immunotherapy. Compared to other solid tumors, indeed, many immunotherapy approaches drastically fail when applied to pancreatic cancer patients. However, thanks to intensive worldwide research focused on this deadly disease, in the last decade our knowledge has greatly increased, which has paralleled an increase in 5-year survival rate to 10% at least in the USA. The scientific community has developed sophisticated tools to study the biology of pancreatic cancer such as Avatars, genetically engineered mice, and organoids, in which novel therapeutic drugs can be tested. Genetically engineered mouse models represent the best choice for studying antitumor immune responses and novel immunotherapy approaches, as they mimic the complex relationship between the immune system and tumor cells. Of equal importance is the possibility to develop novel clinical studies and to enroll patients in clinical trials.

Therefore, our title better refers to the famous Pink Floyd album, “The Dark Side of the Moon”, which experimented with multiple sounds to create this masterpiece. The huge increase in funds focused on PDAC, together with advanced efforts by the scientific community, can provide hope for patients affected by this tumor.

## Declarations

### Copyright

© The Author(s) 2020.
